# University of Virginia

**Published:** 1842-09-24

**Authors:** 


### THE MEDICAL EXAMINER.

PHILADELPHIA, SEPTEMBER 24, 1842.

### MISCELLANEOUS NOTICES.

*University of Virginia.* The Medical Faculty of the University of Virginia has recently sustained a loss by the death of Professor JOHN P. EMMET, who for a long period occupied the chair of Chemistry. The chairs of Medicine and of Anatomy and Physiology continue to be ably filled by Professors HENRY HOWARD and JAMES L. CABELL. By the catalogue for the current year, it appears that thirty-nine students attended the course of Medicine, and forty-two that of Anatomy and Surgery, at the last session.

